# A Case of Graves’ Disease in a Patient with Kartagener’s Syndrome (Complete Visceral Inversion, Chronic Sinusitis, and Bronchiectasis)

**DOI:** 10.70352/scrj.cr.24-00437

**Published:** 2025-01-31

**Authors:** Naoyoshi Onoda, Masashi Yamamoto, Hiroo Masuoka, Minoru Kihara, Takuya Higashiyama, Akihiro Miya, Kahoru Nishina, Akira Miyauchi

**Affiliations:** 1 Department of Surgery, Kuma Hospital, Kobe, Hyogo, Japan; 2 Department of Anesthesiology, Kuma Hospital, Kobe, Hyogo, Japan

**Keywords:** Kartagener’s syndrome, situs inversus totalis, recurrent laryngeal nerve, thyroid surgery, intraoperative neuromonitoring

## Abstract

**INTRODUCTION:**

Kartagener’s syndrome (KS) is a rare disease characterized by a triad of situs inversus totalis, chronic sinusitis, and bronchiectasis. The disorder is caused by a hereditary genetic abnormality that impairs ciliary movement. Although aberrant pass course of the inferior laryngeal nerves due to visceral inversion should be considered during thyroid surgery in patients with KS, no report of surgical treatment for Graves’ disease (GD) in patients with KS has been found to date.

**CASE PRESENTATION:**

A Japanese male in his 40s was referred to our hospital for surgical treatment for drug-refractory GD. He was diagnosed to have KS by genetic alteration of the *DNAH5* gene as well as clinical triad. No abnormal branching in the mediastinal great vessels was identified in the present case, and left-sided non-recurrent inferior laryngeal nerve (NRLN) was not observed during surgery. Previous literature has demonstrated that the presence of a right-sided aortic arch and an anomalous branch of the left subclavian artery, as well as the absence of a left ductus arteriosus demonstrable on preoperative imaging studies, are prerequisites for the development of the extremely rare left-sided NRLN.

**CONCLUSION:**

We reported the first case of surgical treatment for GD in a patient with KS and discussed the preoperative diagnosis of NRLN.

## Abbreviations

KS
: Kartagener’s syndrome
SIT
: situs inversus totalis
PCD
: primary ciliary dyskinesia
GD
: Graves’ disease
TRAb
: TSH receptor antibody
RLN
: recurrent laryngeal nerve
DA
: ductus arteriosus

## INTRODUCTION

Kartagener’s syndrome (KS) is a disease characterized by complete visceral inversion (situs inversus totalis [SIT]), chronic sinusitis, and bronchiectasis.^1,2)^ Although Siewart first described this condition in 1904, Kartagener reported four cases with the above triad in 1933.^2)^ KS is one form of primary ciliary dyskinesia (PCD), a rare autosomal recessive genetic disorder, in which ciliary movement is impaired and the respiratory system becomes susceptible to infection.^3,4)^ In approximately half of the patients with PCD, SIT due to ciliary dyskinesia during embryonic development was recognized.^1)^ KS is estimated to occur in 1 in 30000 births.^2)^

No report of surgical treatment for Graves’ disease (GD) in patients complicating with KS has been found to date. Here, we reported the first case of GD that occurred in a patient with KS. We discussed points to be considered for thyroid surgery in cases of KS, for example, aberrant course of the inferior laryngeal nerve.

## CASE PRESENTATION

A Japanese male in his 40s was referred to our hospital for surgical treatment of GD. The patient was diagnosed with GD 15 years ago and had been treated with antithyroid drugs. One year ago, the hyperthyroid state recurred and was difficult to control, even with dose accelerations. At the time of the first visit, he was taking 20 mg of thiamazole, 50 mg of potassium iodide, 2.5 mg of bisoprolol, 40 mg of telmisartan, 10 mg of ezetimibe, 200 mg of clarithromycin, and 10 mg of montelukast sodium.

He was diagnosed with SIT and chronic sinusitis at age 0. He had undergone surgery for chronic sinusitis 7 years ago and had ablation for atrial fibrillation. He was told to have impaired sperm motility.

His elder brother was taking medicines for GD. His father had a history of lung cancer. There were no SIT or PCD cases in his family. There was no intra-family marriage.

**Fig. 1:**
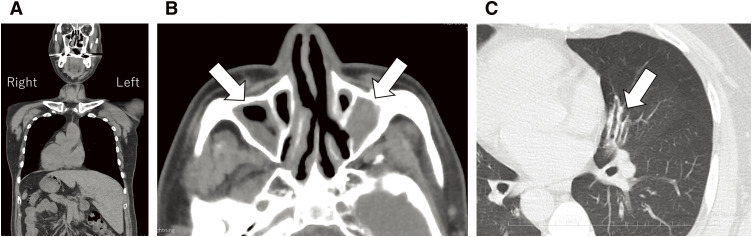
CT demonstrated complete visceral inversion (situs inversus totalis) (**A**). Mucosal thickening and fluid collection were observed in the sinuses (**B**). Ectasias in several bronchus was also observed (**C**).

**Fig. 2:**
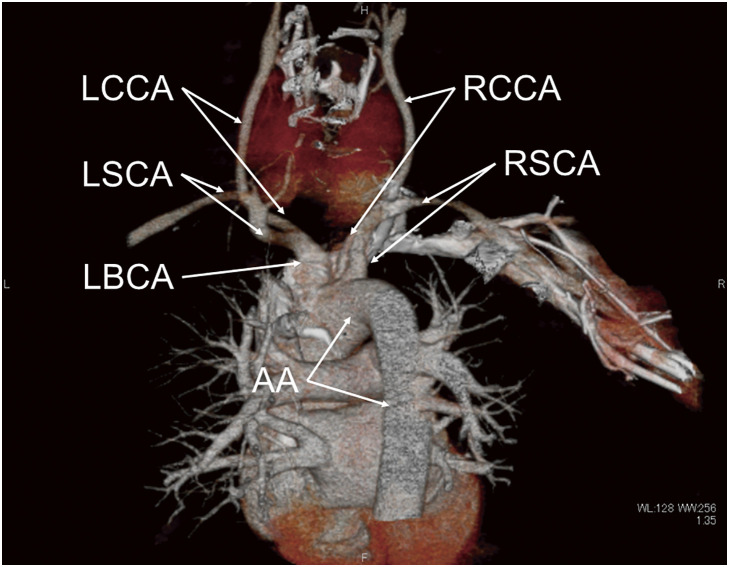
A posterior view of the thoracic great vessels. The RSCA and RCCA branched independently from the AA on the right side. There was no independent branch of the LSCA on the left side, and the LCCA and LSCA branched from the LBCA. AA, aortic arch; LSCA, left subclavian artery; LBCA, left brachiocephalic artery; RSCA, right subclavian artery; RCCA, right common carotid artery; LCCA, left common carotid artery

He was 178 cm high, weighed 95 kg, had a blood pressure of 128/83 mmHg, and a regular pulse rate of 64/min. A diffuse goiter was observed in his neck. Blood examination revealed free T3 of 4.97 (normal range: 2.3–4) pg/mL, free T4 of 0.96 (0.9–1.7) ng/dL, thyroid stimulating hormone (TSH) was below the lower limit, and TSH receptor antibody (TRAb) was as high as 186 (<2) IU/L. No abnormal results were found in blood counts. The hepatic and renal function were within normal range. Chest X-ray demonstrated a right-sided heart. A neck ultrasound revealed diffuse enlargement of the thyroid gland, and the estimated weight was 151 g. CT demonstrated SIT both in the chest and abdomen (**Fig. 1A**). Mucosal thickening and fluid collection were observed in the sinuses (**Fig. 1B**). Ectasias in several bronchi was also observed (**Fig. 1C**). No abnormal branching of the great thoracic vessels was observed. The subclavian artery and common carotid artery branched independently from the aortic arch on the right side. No independent branching of the subclavian artery was observed on the left side. The common carotid artery and subclavian artery branched from the left brachiocephalic artery (**Fig. 2**). Vital capacity measured 4.94 L (106.2%). In comparison, percent predicted forced expiratory volume in 1 second (FEV1.0) was 2.61 L (67.2%), indicating moderate obstructive disorder. Genetic testing for mutations in the *DNAI1* and *DNAH5* genes was performed with the patient’s consent. A pathogenic variant from CGA (Arg) to TGA (Ter) was found in Exon 34 Codon 1883 of the *DNAH5* gene.^5)^

**Fig. 3:**
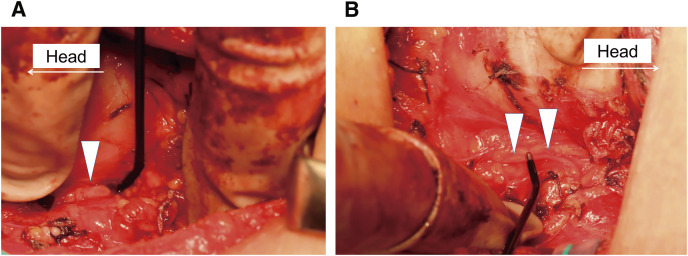
The right inferior laryngeal nerve ran attached to the tracheal wall (**A**: arrowhead), and the left RLN ran obliquely on the dorsal surface of the left lobe, slightly apart from the tracheal wall (**B**: arrowheads). RLN, recurrent laryngeal nerve

A diagnosis of drug-refractory GD associated with KS was made and total thyroidectomy was performed. The thyroid gland was diffusely enlarged and had abundant blood flow. The right inferior laryngeal nerve (recurrent laryngeal nerve [RLN]) was attached to the tracheal wall within the tracheoesophageal groove, and the left inferior laryngeal nerve was obliquely routed along the dorsal surface of the left lobe and slightly separated from the tracheal wall. Neither nerve ran as non-recurrent manner (**Fig. 3A** and **3B**). Intraoperative nerve monitoring showed that the latency from stimulations of the vagus nerve to the vocal cord movement was slower on the right side (6.7 × 10^–3^ seconds) than on the left side (4.7 × 10^–3^ seconds), indicating that the RLN had a longer course on the right side.

Histologically, the thyroid gland was composed of follicles of different sizes, the follicular epithelium was slightly swollen and tall, and absorption vacuoles were seen in the colloid near the follicular epithelium, consistent with GD.

Postoperative fiberoptic laryngoscopy revealed transient left RLN paralysis, which resolved within 1 month after surgery. The patient was discharged uneventfully and is undergoing outpatient treatment with 125 μg/day of levothyroxine supplementation.

## DISCUSSION

KS demonstrates a triad of symptoms, including SIT, chronic sinusitis, and bronchiectasis, and is known as a subtype of PCD caused by mutations in genes that coded the dynein arms of cilia.^3,4)^ The main symptoms of PCD are caused by ciliary dyskinesia in the respiratory system. Patients with KS often suffer from sinusitis and otitis media.^2)^ Other symptoms seen in patients with KS include infertility, as seen in this case, hydrocephalus, anosmia, and visual impairment.^2)^ A link between PCD and parenchymal fatty degeneration of the liver has also been suggested.^6)^ On the other hand, the etiology of GD is excessive production of thyroid hormones due to the appearance of autoantibodies that stimulate thyroid hormone production, such as TRAb. Therefore, it is difficult to assume a causal relationship between the onset of GD and ciliary motility disorder caused by KS. To date, no cases of GD combined with KS have been reported, and the combination of GD in this case was considered coincidental. KS is known to be inherited in an autosomal recessive manner,^2)^ but there was no family member involved with KS in this case. However, at the same time, the fact that GD developed in his brother, who did not have KS, may indicate the involvement of genetic factors in the occurrence of GD in the present case.

When performing surgery in cases of SIT, it is necessary to be aware of the mirror image, which is the opposite of the normal positional relationship. Since the thyroid gland is an almost bilaterally symmetrical organ, there is no need to focus too much on the mirror image. However, SIT patients are often accompanied by congenital abnormalities in the branching of the mediastinal great blood vessels, which can cause abnormalities in the running course of the RLN, so it is particularly important to be aware of the presence of nonrecurrent RLN. Right-sided nonrecurrent RLN is observed at a frequency of 0.3%–0.8% and is well known to be mainly caused by an abnormal branching of the right subclavian artery to the posterior esophagus.^7,8)^ On the other hand, left-sided non-recurrent RLN is extremely rare.^9)^ This is because, in addition to a right-sided aortic arch, an anomalous branch of the left subclavian artery that circles behind the trachea and the absence of a left ductus arteriosus (DA) are essential conditions for its development.^10)^ Masuoka et al. clearly demonstrated that a crucial point in the identification of a left non-recurrent RLN is to determine the left-sided DA in cases of the right-sided aortic arch by identifying a lack of so-called Kommerell diverticulum at the point at which the left subclavian artery originates from the descending aorta.^9,11)^ In this case, although SIT was accompanied and a right-sided aortic arch was observed, no abnormal branching of the large mediastinal vessels was observed. As a result, although the running pattern of the RLN was reversed on the left and right sides, left-sided non-recurrent RLN was not observed.

Since the latency of intraoperative neuromonitoring reflects the distance from the stimulation site to the vocal cord, it has been reported that checking the difference in latency between the left and right sides is useful for intraoperative diagnosis of nonrecurrent RLN. In the nonrecurrent RLN, the latency is clearly shortened because the nerve length is shorter than normal RLN.^12)^ Using a similar principle, we electrophysiologically demonstrated that the right RLN, in this case, was longer than the left, as the latency during stimulation of the right vagus nerve was unusually longer than that of the left.

KS is susceptible to infections, particularly in the respiratory system.^2)^ The patient suffered from transient left vocal cord palsy, as well as he had bronchiectasis with moderate obstructive respiratory dysfunction. We, thus, took intimate care of his mis-swallowing during the early period after surgery. Fortunately, no respiratory complications were identified postoperatively.

## CONCLUSION

To our knowledge, this is the first report of surgical treatment of GD in a patient with KS. Although extremely rare, when performing thyroid surgery in cases with situs inverts such as found in this syndrome, it is necessary to be aware of the presence of a left-sided non-RLN by checking for the presence of an anomalous branch of the left subclavian artery and the absence of a left DA. Furthermore, comparing the latency between the left and right sides by intraoperative nerve monitoring may help detect abnormally short nerve lengths of non-RLNs.

## ACKNOWLEDGMENTS

We thank Mr. Takeo Kawai for his contributions in identifying genetic abnormalities in the *DNAI1* and *DNAH5* genes in the patient.

## DECLARATIONS

### Funding

The authors did not receive any financial support for this article.

### Authors’ contributions

NO was the patient’s attending surgeon, who collected the patient’s information, wrote the original draft, and reviewed and edited the manuscript.

MY, HM, MK, TH, AMiya, and KN reviewed and edited the manuscript.

AMiyauchi supervised, reviewed, and edited the manuscript.

### Availability of data and materials

Not applicable.

### Ethics approval and consent to participate

Not applicable.

### Consent for publication

Consent for publication was obtained from the patient.

### Competing interests

The authors declare that they have no conflicts of interest.
